# Antifungal Patterns of Dermatophytes: A Pathway to Antifungal Stewardship in Eastern India

**DOI:** 10.7759/cureus.64479

**Published:** 2024-07-13

**Authors:** Satyendra P Yadav, Manoj Kumar, Kumari Seema, Abhay Kumar, Manju Boipai, Prabhat Kumar, Ashok K Sharma

**Affiliations:** 1 Microbiology, Phulo Jhano Medical College and Hospital, Dumka, IND; 2 Microbiology, Rajendra Institute of Medical Sciences, Ranchi, IND; 3 Dermatology, Rajendra Institute of Medical Sciences, Ranchi, IND

**Keywords:** tinea, resistance, antifungal sensitivity, trichophyton, dermatophytes

## Abstract

Background

Dermatophytosis is a superficial fungal infection caused by a group of pathogenic keratinophilic fungi. The increase in the incidence of superficial fungal infections, combined with the emergence of antifungal resistance, represents both a global health challenge and a considerable economic burden. Recent years have witnessed a surge in dermatophytosis cases, accompanied by the emergence of antifungal-resistant strains. This study aimed to analyze the in vitro antifungal susceptibility patterns and determine the minimum inhibitory concentrations (MIC) of antifungal drugs among isolated species using the broth microdilution method.

Methodology

This cross-sectional study was conducted between September 2021 and August 2022. Patients with symptoms or clinical features of fungal infection, including skin, hair, and nail lesions indicative of Tinea infections, were included. Samples underwent processing, including potassium hydroxide (KOH) mounting, direct microscopic examination, and culture on Sabouraud Dextrose Agar (SDA) with antibiotics. Antifungal susceptibility testing was subsequently conducted.

Results

Trichophyton mentagrophytes emerged as the most common isolate among patients with Tinea infections. MIC values of various drugs were analyzed, with itraconazole exhibiting a minimum MIC of 0.03 µg/ml and a maximum of 0.50 µg/ml. Terbinafine showed an MIC of 0.010 µg/ml and a maximum of 1.00 µg/ml. Ketoconazole had a minimum MIC of 0.03 µg/ml and a maximum of 0.50 µg/ml. Fluconazole exhibited a minimum MIC of 0.10 µg/ml and a maximum of 1.00 µg/ml. Lastly, miconazole demonstrated a minimum MIC of 0.03 µg/ml and a maximum of 2.00 µg/ml.

Conclusion

Accurate diagnosis is crucial for fungal infections to enable early treatment and reduce transmission. With an increasing trend in resistance among dermatophytes, there is a growing need to conduct susceptibility testing of antifungal agents, particularly in cases of long-term infections, recurrent infections, and individuals who do not respond to medication.

## Introduction

Dermatophytes are fungi that cause superficial infections of the skin, hair, and nails, known as dermatophytosis or ringworm. Tinea, as it is clinically referred to, can manifest in various sites: Tinea capitis (scalp), Tinea corporis (body), Tinea cruris (groin), Tinea pedis (feet), Tinea barbae (beard area), Tinea mannum (hands), Tinea faciei (face), and Tinea unguium (nails) [1].

Dermatophyte infections spread through direct contact with humans, animals, soil, or contaminated objects. Common pathogens include Microsporum, Trichophyton, and Epidermophyton species [2-4]. The increase in the incidence of superficial fungal infections combined with the emergence of antifungal resistance represents both a global health challenge and a considerable economic burden. A prevalence of 6.09% to 27.6% has been reported in studies from South India, while a high prevalence of 61.5% has been recorded in North India [5]. The management of dermatophytosis poses a considerable challenge due to factors such as limited treatment options, emerging drug resistance, and inadequate surveillance systems. Antifungal stewardship, which involves the rational use of antifungal agents to optimize patient outcomes while minimizing the development of resistance, has become imperative in addressing these challenges. The estimated lifetime risk of dermatophyte infection is 10-20% [6-8].

In Eastern India, where dermatophytosis is endemic, there is very little data available from Eastern and Gangetic India [9]. There is a need to understand the antifungal susceptibility patterns of dermatophytes circulating in the region. Such knowledge is essential for guiding empirical therapy, selecting appropriate antifungal agents, and monitoring trends in resistance. Additionally, establishing local epidemiological data and clinical breakpoints for antifungal agents specific to Eastern India can enhance the effectiveness of antifungal stewardship initiatives. Treatment involves topical or oral antifungal drugs, with systemic therapy required for severe cases like tinea capitis and tinea unguium. Topical antifungal medications like ketoconazole, ciclopirox, tolnaftate, clotrimazole, amorolfine, econazole, and terbinafine are commonly used for dermatophyte infections due to their demonstrated efficacy. However, severe and long-term infections like tinea capitis and tinea unguium require systemic antifungals such as itraconazole, terbinafine, and griseofulvin [10-12]. Newer systemic agents like posaconazole and ravuconazole show promise in managing chronic dermatophytosis and are under evaluation [13].

Therefore, this study aims to investigate the antifungal pattern of dermatophytes in Eastern India, shedding light on the prevalence of dermatophyte species and their susceptibility profiles to commonly used antifungal agents. By understanding the clinico-epidemiological aspects of dermatophytosis in the region, this study helps contribute valuable insights into the optimization of antifungal therapy and the development of evidence-based antifungal stewardship strategies.

## Materials and methods

Study setting and participants

The study was conducted at the Department of Microbiology, Rajendra Institute of Medical Sciences, Ranchi, from September 2021 to August 2022. Ethical clearance was obtained from the Institutional Ethics Committee. A total of 100 cases diagnosed with clinically suspected superficial fungal infections of the hair, skin, and nails, referred from the Dermatology department, were included.

Inclusion criteria

Patients presenting with symptoms including itching, scaling, dryness, erythema, and lesions with central clearing surrounded by redness were enrolled in the study. Demographic information, occupation, medical history, family history, etc., were documented using a case record form. Informed consent was obtained from either the patient or their attendant prior to sample collection.

Sample collection

Skin scrapings were collected from the edges of the lesions located on the scalp, groin, perineal and perianal regions, armpit, and nails. Hair clippings were obtained, and infected hairs were gently plucked from the scalp, hands, and feet, ensuring the removal of a minimum of 10 hairs. Scales were scraped off from the affected areas, while infected nail samples were obtained by scraping the affected nail area.

Potassium hydroxide (KOH) mount

Skin scale specimens underwent direct microscopic examination using a 10% KOH wet mount, while hair and nail specimens were examined using 40% KOH. Furthermore, samples were cultured on both Sabouraud Dextrose Agar (SDA) and Dermatophyte Test Medium (DTM) to facilitate fungal growth.

Culture

The samples were cultured on SDA and DTM.

Antifungal susceptibility testing

After sub-culturing on a nutritionally deficient medium called Potato Dextrose Agar, antifungal susceptibility testing of dermatophytes (M-38A) was performed using the microbroth dilution method at the Department of Medical Microbiology, Post Graduate Institute of Medical Education and Research (PGIMER), Chandigarh. Cases of dermatophytosis were analyzed using the broth microdilution method, as recommended by the Clinical and Laboratory Standards Institute, and modified for dermatophytes, to determine susceptibility against antifungals including fluconazole, ketoconazole, miconazole, itraconazole, and terbinafine [14]. Stock solutions of the drugs were prepared, followed by stepwise dilution. The minimum inhibitory concentration (MIC) 50 was determined as the concentration of the drug that inhibited 50% of the isolates, while the MIC90 was based on the drug concentration that inhibited 90% of the isolates [15].

Statistical analysis

All samples were analyzed using Microsoft Excel and SPSS software version 21. Mean and SD were calculated for MIC values. Proportions and percentages were used as appropriate, depending on the nature of the data obtained for the study. Sensitivity for the antifungal drugs was calculated.

## Results

**Figure 1:**
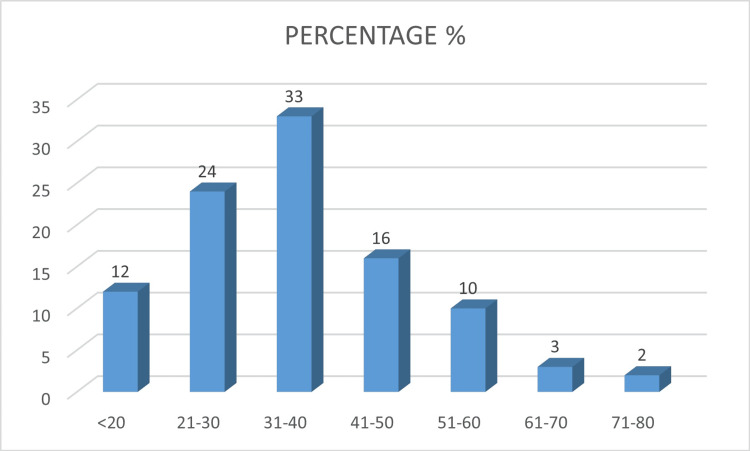
Age-wise distribution of dermatophytes (N=100): X-axis represents age in years, Y-axis represents percentage.

Among the 100 patients, the majority of samples were collected from skin scrapings (98%), while 1% came from nails, and 1% from hair follicles. The ages of patients ranged from 14 to 75 years, with a mean age of 35.72 ± 13.611. Females (n=22) had a mean age of 38.04 ± 13.05, and males (n=78) had a mean age of 35.06 ± 13.68. The age group with the highest number of dermatophytosis cases was 31-40 years, as shown in Figure 1.

Distribution of lesions as per clinical features

**Figure 2:**
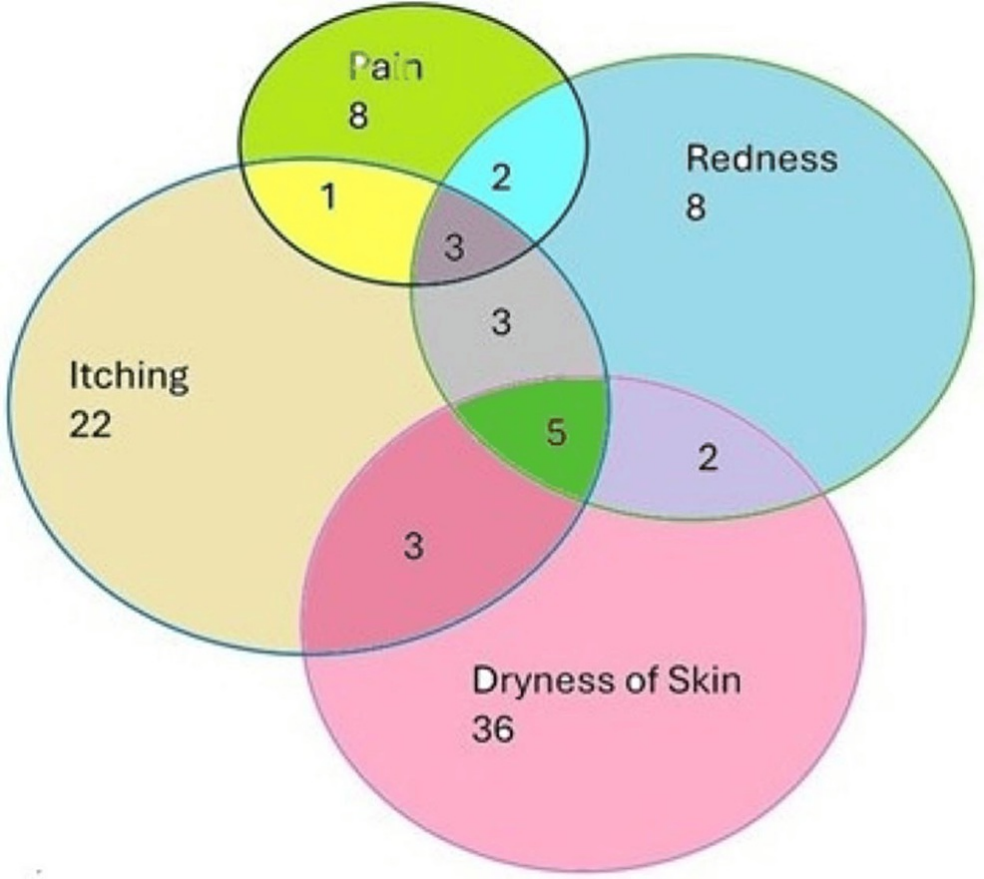
Venn diagram showing the distribution of patients based on clinical features.

The study of 100 patients with dermatophytosis revealed varied clinical features. The most common symptoms were dry skin (34%), itching (19%), and itching with redness (15%). Other symptoms included redness without itching (8%), itching with dry skin (5%), and itching with redness and liquid secretion (3%). Less common symptoms included itching with dryness or pain and redness (3% each), pain with redness (2%), darkening of the skin (1%), and severe itching (1%), as detailed in Figure 2.

Distribution as per clinical diagnosis

**Figure 3:**
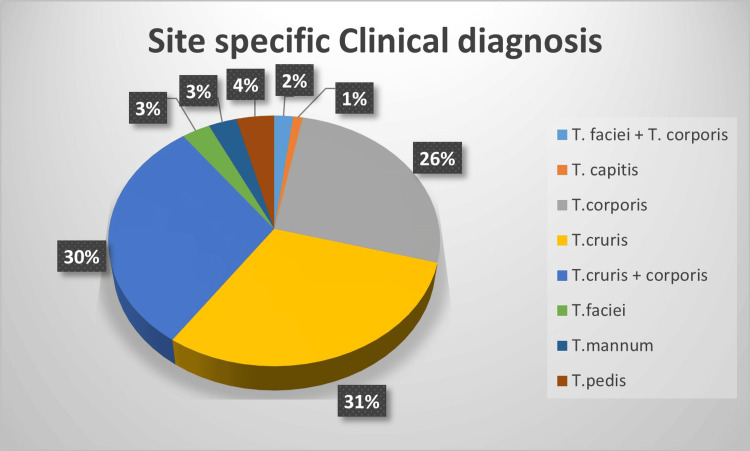
Skin lesions showing site-specific clinical diagnoses.

The distribution of clinical diagnoses among the 100 patients revealed various forms of dermatophytosis. The most common diagnosis was Tinea cruris, observed in 30% of patients, followed by Tinea corporis, also at 30%. Combined infections such as Tinea cruris with corporis were found in another 30% of cases. Tinea pedis was diagnosed in 4% of patients, while Tinea faciei, Tinea capitis, Tinea mannum, and combinations like Tinea faciei with corporis were less prevalent, each representing 2-3% of cases (Figure 3).

Distribution as per species identification

**Figure 4:**
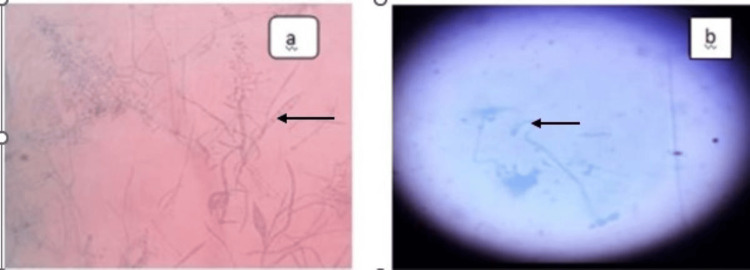
Microscopic views of Lactophenol Cotton Blue (LPCB) mount of specimens. (a) LPCB mount showing pencil-shaped macroconidia and thin septate hyphae of *T. rubrum*. (b) LPCB mount showing thin septate and spiral hyphae of *T. mentagrophytes*.​​​​​​​

On SDA culture, 51% of the samples were sterile, 25% were contaminated, and dermatophyte growth was observed in 24%. Among the positive cultures, the species identified were based on culture characteristics, morphological colony appearance, and microscopic findings in Lactophenol Cotton Blue (LPCB) mount. *Trichophyton mentagrophytes* was the most prevalent, found in 71% of positive cultures, followed by *T. rubrum* at 25%. Mixed infections involving *T. mentagrophytes* and Microsporum were observed in 4%. Species identification was achieved through LPCB mount of the specimen, as illustrated in Figure 4.

Antifungal susceptibility testing

Common drug resistance was observed in *T. mentagrophytes* and *T. rubrum*, as shown in Table 1. *Microsporum sp.* was sensitive to all drugs. Multidrug resistance was observed in two strains of *T. mentagrophytes*, and *T. rubrum* showed resistance to only one drug, miconazole.

**Table 1:** Species-wise drug susceptibility (S = Sensitive, R = Resistant).

Drug	*T. mentagrophytes* (N=17)	*T. rubrum* (N=6)	Microsporum (N=1)
Ketoconazole	R=2	S	S
Fluconazole	R=4	S	S
Itraconazole	S	S	S
Miconazole	R=4	R=1	S
Terbinafine	S	S	S

Table 2 summarizes the drug sensitivity percentages and the mean and SD of MIC values for five antifungal drugs. Itraconazole demonstrated 100% sensitivity, with a mean MIC of 0.1960 µg/ml and an SD of 0.177614. Terbinafine followed closely with 95.83% sensitivity, a mean MIC of 0.3131 µg/ml, and an SD of 0.226225. Ketoconazole exhibited 91.66% sensitivity, with a mean MIC of 0.0950 µg/ml and an SD of 0.10023. Fluconazole and miconazole showed sensitivities of 83.33% and 79.16%, respectively, with mean MIC values of 0.2106 µg/ml and 0.3219 µg/ml, and SDs of 0.20160 and 0.45598, respectively.

**Table 2:** Distribution of patients according to drug sensitivity, and mean and SD of numerical outcomes.

Drug	Percentage (%)	Lowest (MIC µg/ml)	Highest (MIC µg/ml)	Mean	SD
Ketoconazole (22)	91.66	0.03	0.50	0.0950	0.10023
Fluconazole (20)	83.33	0.10	1.00	0.2106	0.20160
Itraconazole (24)	100	0.500	0.500	0.1960	0.177614
Miconazole (19)	79.16	2.00	2.00	0.3219	0.45598
Terbinafine (23)	95.83	0.010	1.000	0.3131	0.226225

## Discussion

Our study assessed the susceptibility of dermatophytes to common antifungal drugs, including itraconazole, ketoconazole, fluconazole, miconazole, and terbinafine. Conducted at Rajendra Medical College and Hospital, Ranchi, Jharkhand, the research aimed to understand the antifungal sensitivity patterns among patients with suspected dermatophytosis.

Our epidemiological analysis revealed a higher prevalence of dermatophytosis in males (78%) compared to females (22%), possibly due to increased exposure and physical activity. The mean age of affected individuals was 35.72 years, with the most common occurrence observed within the 31-40 age range. Clinically, *T. cruris* (30%) and *T. corporis* (26%) were the most frequently diagnosed conditions, aligning with previous findings [16].

This study assessed the susceptibility of dermatophyte strains to five antifungal drugs. Terbinafine demonstrated the lowest MIC at 0.01 μg/mL, followed by miconazole, ketoconazole, and itraconazole at 0.03 μg/mL, and fluconazole at 0.1 μg/mL. Consistent with Sharma et al., griseofulvin had a minimum MIC of 0.1 μg/mL, while itraconazole and terbinafine followed. Fluconazole showed the highest MIC across all isolated species. Similarly, Araujo et al. reported lower MICs for itraconazole and terbinafine compared to fluconazole [17].

In Tahiliani S et al.'s study, higher MIC values were reported for terbinafine against both *T. mentagrophytes* and *T. rubrum* (0.256 μg/mL) [18]. Bueno JG et al. found terbinafine to have the lowest MICs against dermatophytes, closely followed by voriconazole [19]. In our study, the maximum observed MIC was for fluconazole at 0.1 μg/mL, contrasting with other studies where fluconazole's highest MIC reached 32 μg/mL [20]. Maurya VK et al. reported 38.6% of fungal isolates with a fluconazole MIC of 4 μg/mL and 6.6% with the highest MIC of 64 μg/mL [21]. Additionally, itraconazole exhibited low MIC values (≤0.125 μg/mL) in 90.6% of isolates, while ketoconazole showed MICs of ≤0.5 μg/mL in all isolates. However, terbinafine showed higher MICs, with 21.33% of isolates at 2 μg/mL and 29.33% at 4 μg/mL [22].

The high level of terbinafine sensitivity observed in this study may be attributed to the lower virulence of the dermatophyte species present, possibly influenced by external factors such as heat and humidity. Additionally, pharmacological properties of the drug may have also contributed to its high sensitivity. It is worth noting that *Trichophyton indotineae*, a newly identified dermatophyte species found in near-epidemic form on the Indian subcontinent, could not be isolated in our study. This fungus is identical to genotype VIII within the *T. mentagrophytes*/*T. interdigitale* species complex, which was identified in 2019 through sequencing the internal transcribed spacer (ITS) region of ribosomal DNA of the dermatophyte [23].

In conclusion, our study elucidated the antifungal susceptibility patterns of dermatophytes, highlighting terbinafine's notable efficacy. The prevalence of dermatophytosis skewed towards males, with *T. cruris* and *T. corporis* being the most diagnosed conditions. Our findings contribute to the understanding of dermatophyte susceptibility in clinical settings, emphasizing the need for continued surveillance and research in this area.

Limitations of the study include the inability to isolate the *Trichophyton indotineae* species based on cultural characteristics. Phenotypic characteristics and molecular characterizations were not performed due to limited resources. Additionally, the low positivity rate in sample cultures limits the generalizability of the prevalence of different species on a larger scale. Furthermore, the drug resistance standardization of antifungal drugs could not be defined in this area due to inadequate positive samples.

## Conclusions

The accurate diagnosis of fungal infections is important for timely treatment and preventing transmission. With the rise of drug-resistant dermatophytes due to indiscriminate antifungal usage and delayed therapy initiation, there is an urgent need for antifungal susceptibility testing, stewardship, and robust antifungal policies. These measures are crucial for guiding clinicians in empirically prescribing suitable antifungals, particularly in cases of chronic or recurrent dermatophytosis, and in instances of treatment failure or relapse. By implementing these strategies, we can better combat the challenges posed by evolving fungal resistance and improve patient outcomes in the management of dermatophyte infections.
